# Management of Crohn’s Disease and Its Complexities: A Case Report and Literature Review

**DOI:** 10.7759/cureus.67499

**Published:** 2024-08-22

**Authors:** Peddi Pavani, Balakrishna Ravella, Krish Hitesh Patel, Niragh Sikdar, Neeraj Kancherla, Tamer Zahdeh, Resheek Nerella

**Affiliations:** 1 General Surgery, Kurnool Medical College, Kurnool, IND; 2 Internal Medicine, OSF Health St. Mary Medical Center, Galesburg, USA; 3 Internal Medicine, Government Medical College and Hospital, Surat, IND; 4 Medicine, Medical College and Hospital Kolkata, Kolkata, IND; 5 Internal Medicine, Andhra Medical College, Visakhapatnam, IND; 6 Internal Medicine, Hadassah Medical Center, Jerusalem, ISR; 7 Trauma Surgery, University of Illinois College of Medicine at Peoria (UICOMP), Peoria, USA

**Keywords:** intra-abdominal, laparotomy, nf-κβ pathway, ileocecectomy, crohn's disease (cd)

## Abstract

This case report and literature review explore Crohn's disease (CD), a chronic inflammatory disorder of the gastrointestinal tract characterized by unpredictable flare-ups and remissions. The study highlights the diverse clinical manifestations, including abdominal pain, diarrhea, and weight loss, which significantly affect quality of life. It examines the interplay of genetic, environmental, and immune factors in CD's pathogenesis and the complexities in managing the disease. Through a case study of a 20-year-old male, the report addresses various treatment strategies, including medications and surgery, and emphasizes the challenges, particularly post-surgical complications like small bowel leaks. The findings underscore the need for vigilant postoperative care to enhance patient outcomes in this complex condition.

## Introduction

The term "inflammatory bowel disease" (IBD) is used to describe Crohn's disease (CD) and ulcerative colitis which are immunologically mediated inflammatory diseases of the gastrointestinal tract. CD is often diagnosed at a young age and has a chronic course coupled with acute flare-ups. Any part of the gastrointestinal system can be impacted by CD with roughly one-third of patients being involved limited to the small bowel, particularly the terminal ileum; another 20% have involvement limited to the colon; and approximately 50% have involvement to both the colon and small bowel. Most patients go through unpredictable remissions and relapses, and there is no known cure leading to a deficient quality of life [1]. IBD is usually characterized by dysregulation of the nuclear factor kappa B (NF-κβ) pathway and proteins that regulate it, which results in uncontrolled inflammation and impaired immunity. In both CD and UC, there is a rise in the levels of proinflammatory cytokines that are controlled by NF-κβ [2].

In IBD, particularly in CD, the gut microbiota is known to have an impact on how the inflammatory response is developed. Typically, the inflammation is transmural, and biopsies may reveal granulomas with a discontinuous distribution along the longitudinal axis during pathological evaluation. Intestinal fistulas, inflammatory tumors, and intra-abdominal abscesses are some of the irreparable tissue damages caused by this inflammatory process [3], CD flare-ups are commonly accompanied by fever, weight loss, anemia, diarrhea (which may include blood and mucus), flatulence/bloating, and right lower quadrant abdominal pain. Perianal abscess, perianal CD, and cutaneous fistulas are often detected in severe cases [4]. Mild to moderate CD is often treated with drugs such as mesalamine, immunomodulators, and steroids, whereas severe CD requires additional biologic drugs such as infliximab and adalimumab [5]. Patients with CD have a wide range of surgical indications. Given that CD cannot be cured, there must be rigorous guidelines for surgical indications, and the procedure should be as limited and less invasive as feasible. Since restricted bowel resection and strictureplasty are safe and useful, the once-popular bypass procedure has been almost abandoned [6]. Surgery provides more definitive treatment for CD, but it comes with several complications, the most severe of which include wound infections at the surgical site, anastomotic leaks (both acute and chronic), and anastomotic strictures. Anastomotic sinus can result from a persistent anastomotic leak, while an abscess or sepsis can be caused by an acute leak. Reducing postoperative infection problems requires preoperative treatment that includes nutritional support, intravenous (IV) antibiotics, weaning off immunosuppressive drugs, and, if present, percutaneous drainage of abscesses [7]. We present the case of a 20-year-old with CD who had laparoscopic ileocecectomy for small bowel obstruction. Post-surgery, he developed fever and tachycardia, leading to exploratory laparotomy, which found a small bowel leak and significant contamination.

## Case presentation

**Figure 1:**
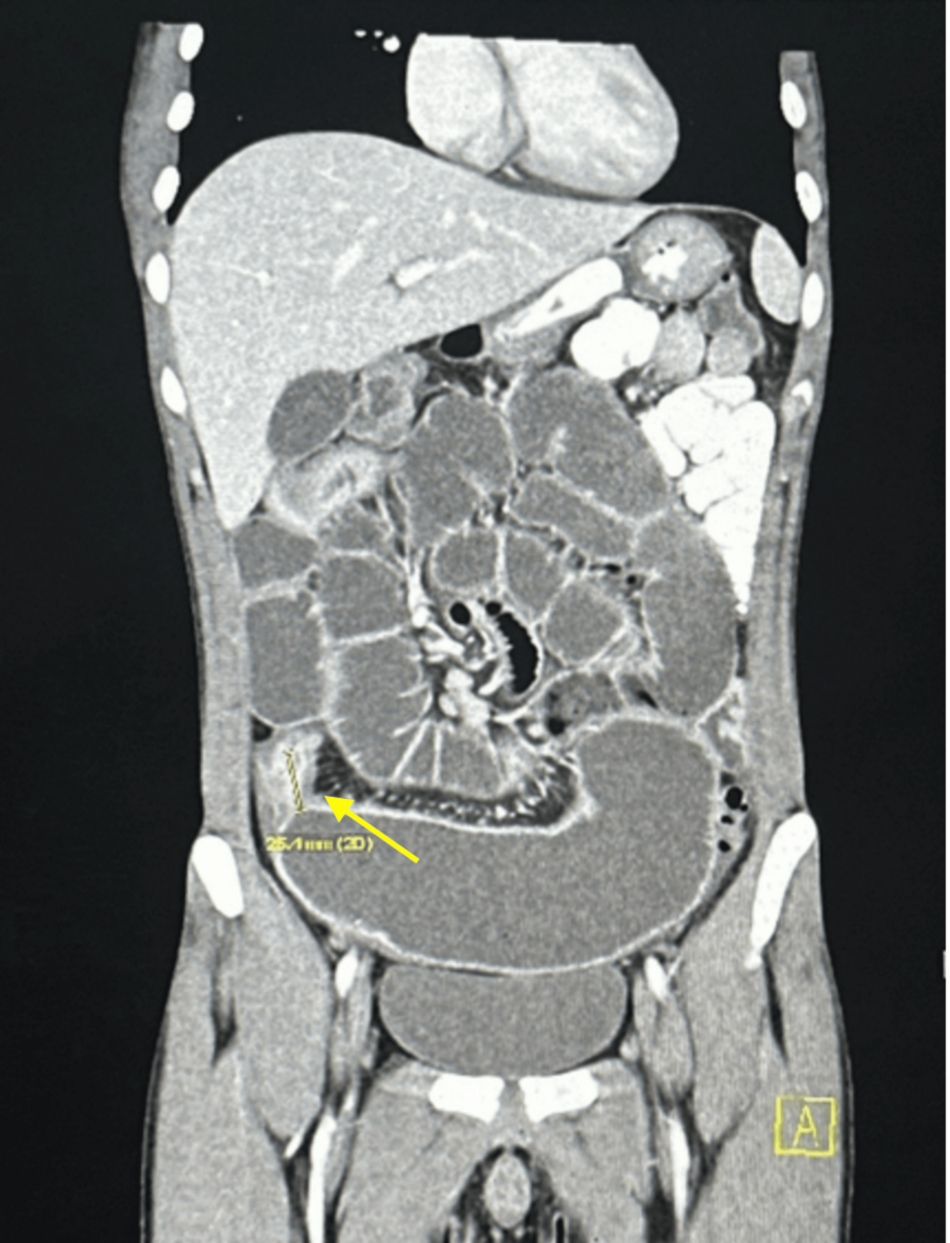
CT abdomen and pelvis showing active inflammatory small bowel Crohn’s disease with luminal narrowing measuring 2.5 cm in length and involving the distal and terminal ileum, with small bowel obstruction

A 20-year-old male with a past medical history of CD presented with abdominal pain. The patient reports progressively worsening cramping, diffuse 8/10 abdominal pain associated with nausea and bloating for the last three days before admission. The rest of his review of systems was unremarkable. His vital signs including temperature were within normal limits. Physical examination was notable for a mildly distended abdomen with minimal tenderness to deep palpation. His family history is significant for CD in his sister. He was recently diagnosed with Crohn’s disease after multiple visits to the emergency department for intermittent cramping abdominal pain with nausea and vomiting. At that time, colonoscopy revealed ileitis with narrowing of the terminal ileum, and he was started on prednisone 35 mg daily and Stelara infusions. Two months before his current admission, a computed tomography (CT) abdomen and pelvis with oral and IV contrast showed gradual dilation of the distal jejunum with markedly dilated loops of ileum measuring up to 5.7 cm as seen in Figure 1.

**Figure 2:**
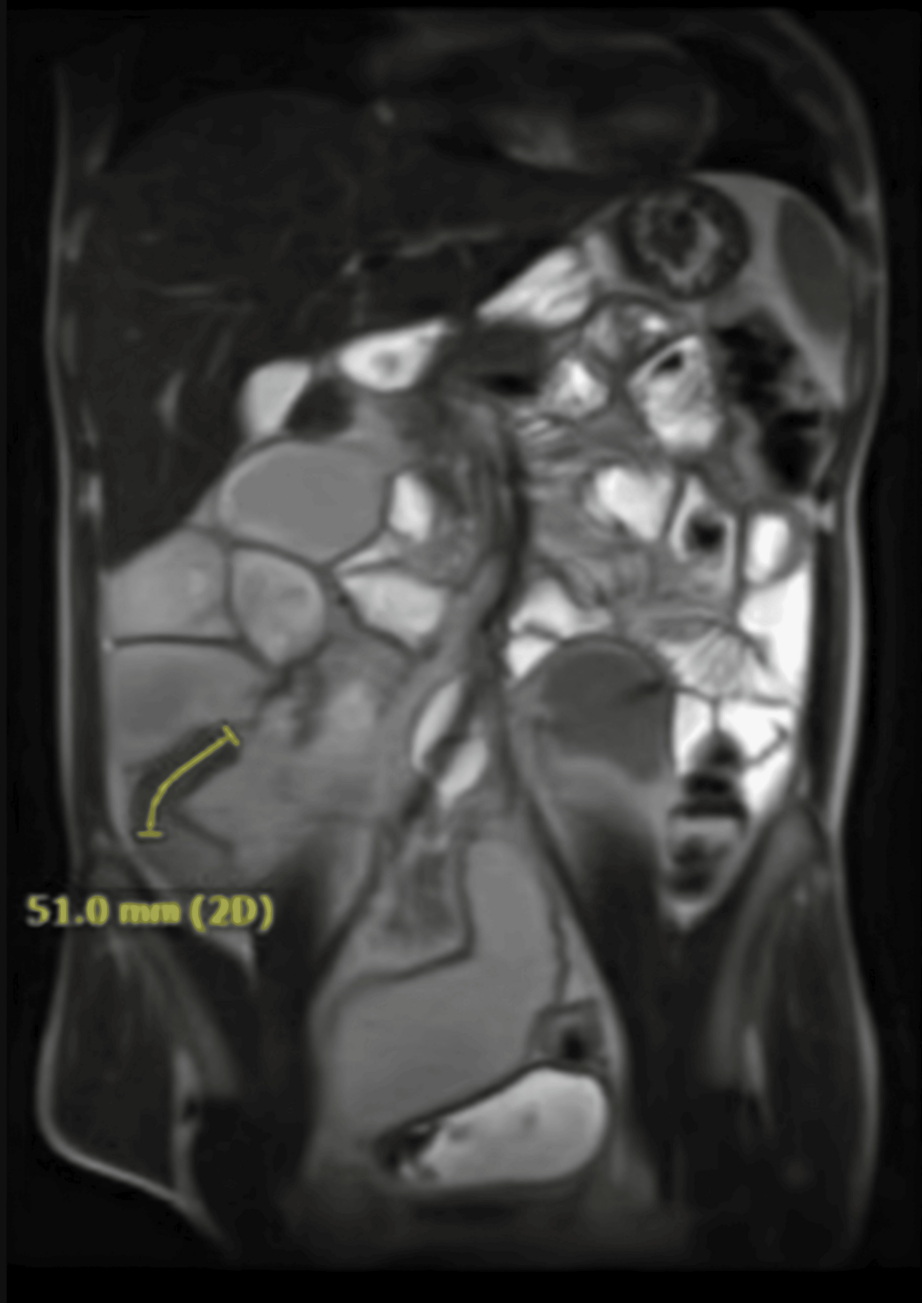
Magnetic resonance enterography (MRE) shows a narrow terminal ileum with associated wall thickening, mucosal hyperenhancement, mildly restricted diffusion, and a markedly dilated upstream small bowel measuring up to 5 cm in caliber

Magnetic resonance enterography (MRE) then also demonstrated actively inflamed and narrowed terminal ileum causing marked upstream dilation, as well as findings suggestive of active inflammation of the dilated small bowel as seen in Figure 2.

Initial blood workup was remarkable for hemoglobin of 9.8 without leukocytosis. CT abdomen and pelvis with contrast revealed a known transition point at the terminal ileum (consistent with the most recent MRE demonstrating chronic stricture) with worsening upstream dilatation measuring at 7.3 cm in diameter and small bowel obstruction. The patient was admitted to the surgery ward and planned for further surgical evaluation for possible ileal resection. Prior to surgical evaluation, he was resumed on IV prednisone 40 mg, and a peripherally inserted central catheter (PICC) line was inserted for total parental nutrition (TPN), which slightly improved his symptoms as he started tolerating pain with having bowel movement and flatus. The patient then underwent laparoscopic ileocecectomy with ileostomy formation rather than anastomosis given the patient’s poor nutrition from chronic obstruction as well as high-dose steroids. Gross examination of the excised specimen (27 cm of ileum and 4 cm of cecum in length) demonstrated extensive ulceration and cobblestone appearance, and the pathology report revealed ulcerative transmural ileitis, consistent with the active phase of reported CD. 

**Figure 3:**
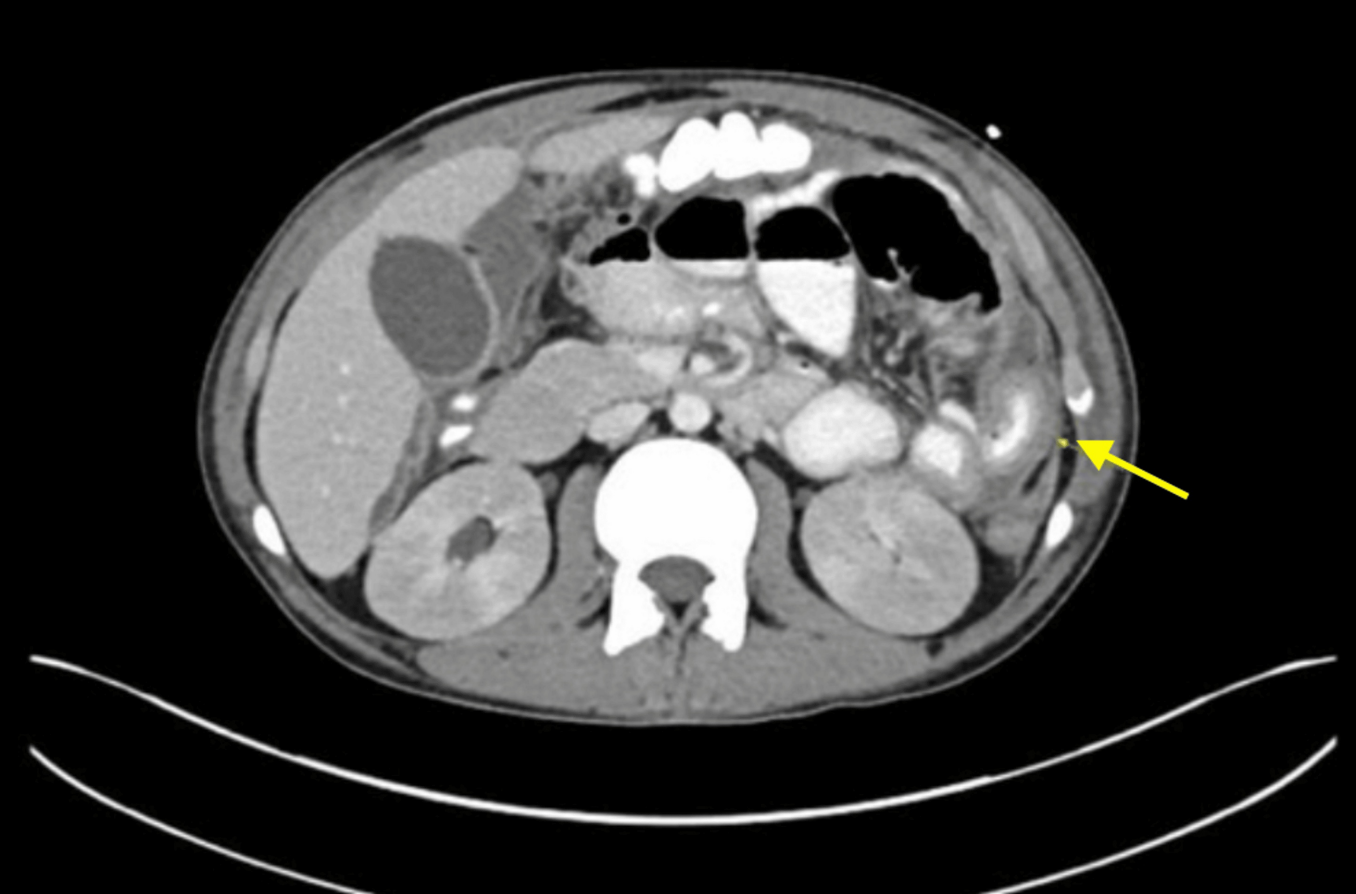
CT abdomen and pelvis with oral contrast showing new circumferential wall thickening and mucosal hyperenhancement of a small segment of patulous ileal bowel loop, as well as contrast leak concerning for small bowel leak

After the procedure, the patient complained of abdominal pain that was controlled with patient-controlled analgesia (PCA). Following that, the patient had an episode of persistent tachycardia and oxygen desaturation down to 88% while on 2 L of continuous nasal cannula oxygen, and labs were significant for worsening leukocytosis close to 20,000. CT chest, abdomen, and pelvis with contrast was notable for a new circumferential wall thickening and mucosal hyperenhancement, which likely reflected active disease or possible superimposed infectious enteritis, for which zosyn and fluconazole were started, and interventional radiology was consulted regarding low ostomy output. Paracentesis was subsequently performed with about 1400 cc of purulent fluid removed. The patient then developed a spike in fever (38.8°C), chills, tachycardia of 140, and worsening leukocytosis up to 23,000. CT abdomen and pelvis with oral and IV contrast was therefore conducted which revealed abdominal extravasation concerning postoperative small bowel leak as seen in Figure 3, and the patient underwent prompt exploratory laparotomy for small bowel evaluation and ileostomy revision. 

**Figure 4:**
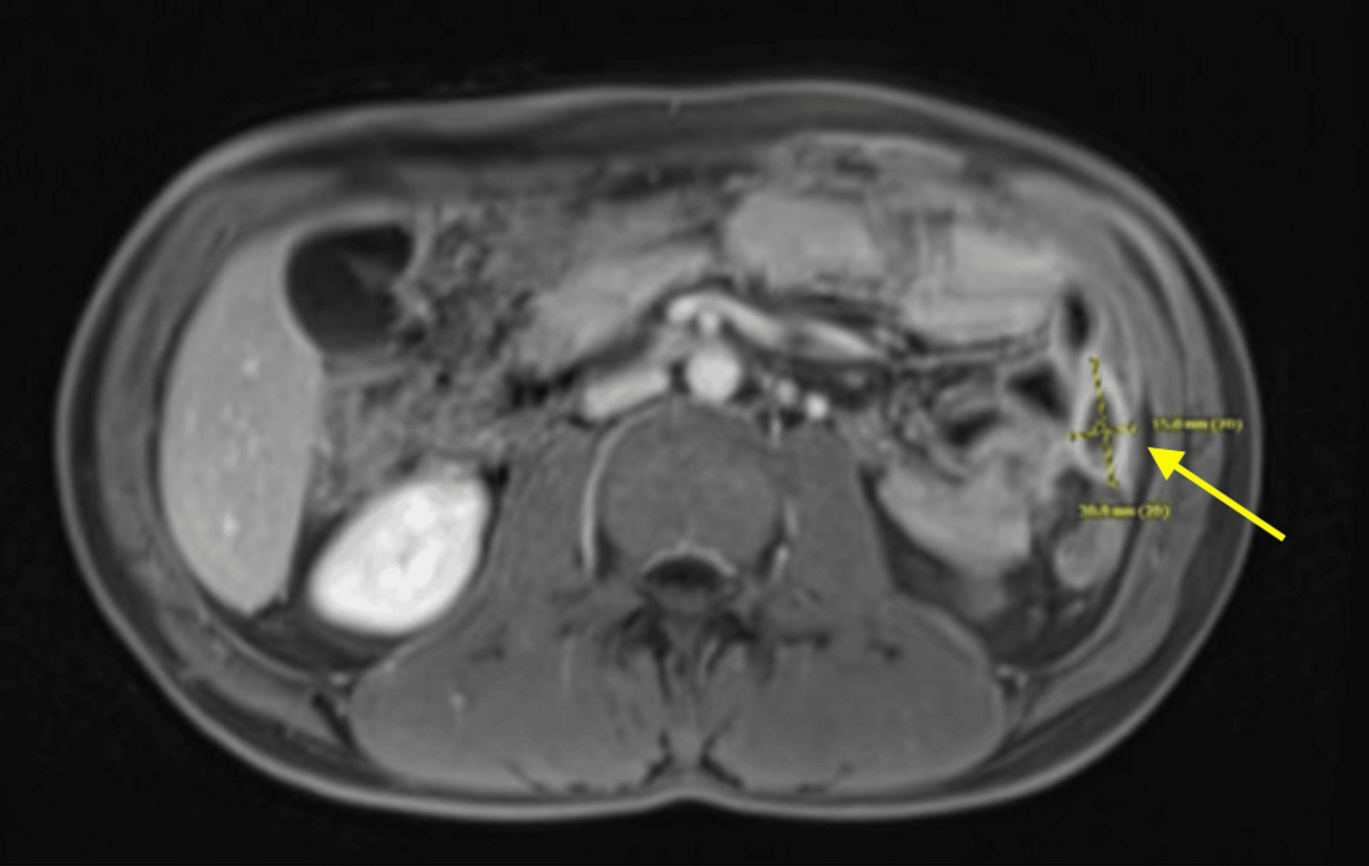
A follow-up MRE showed a 3.1 x 1.5 cm left lateral residual abdomen collection MRE: Magnetic resonance enterography

Exploratory laparotomy revealed a 1 cm staple line dehiscence with small bowel leak, extensive intraperitoneal contamination, and inflamed terminal ileum. The surgeon resected 18.1 cm of bowel and created a new end ileostomy, recommending a six-month wait before reversal. Post-surgery, the patient received a blood transfusion, antibiotics, and fluconazole. CT showed moderate to large ascites with blood products, treated with paracentesis and micafungin for *Candida krusei.* The patient improved gradually and was discharged with outpatient IV antibiotics and ostomy care instructions. A follow-up MRE at two months showed significant reduction in fluid collections as shown in Figure 4. The patient responded well to the treatment given and was scheduled for further outpatient follow-up.

## Discussion

CD, a chronic inflammatory ailment of the gastrointestinal tract, affords an impressive challenge in its control. Epidemiologically, it demonstrates a worldwide distribution with a higher prevalence in Western countries, impacting people across a huge age spectrum [8]. The etiology entails a complex interaction of genetic, environmental, and immune factors, mainly transmural inflammation affecting any part of the gastrointestinal tract [9]. Common signs and symptoms consist of abdominal pain, diarrhea, weight reduction, and fatigue, substantially compromising sufferers' high quality of life [8]. Despite sizeable development in comprehending its pathogenesis, therapeutic alternatives, and surgical interventions, Crohn's ailment remains a multifaceted entity worrying a nuanced technique.

The literature on Crohn's ailment is extensive, underscoring the multifaceted nature of the disease. The ailment's heterogeneity is properly documented, providing various clinical phenotypes, starting from inflammatory to structuring and penetrating forms [10]. The chronic and relapsing nature of CD necessitates a multifaceted therapeutic approach, encompassing immunosuppressive agents, corticosteroids, and biologics [11]. Surgical interventions, together with bowel resection, strictureplasty, and stoma advent, play a pivotal role in managing complications and enhancing the overall quality of life for affected individuals [12]. Despite therapeutic improvements, demanding situations persist in achieving sustained remission and preventing disease recurrence, prompting ongoing exploration into novel therapies, together with personalized remedy strategies and microbiome-based interventions [11,12].

The provided case contributes to the literature by means of offering a unique clinical perspective. The affected person's records of Crohn's ailment, complex via chronic inflammatory modifications inside the terminal ileum, align with present literature emphasizing the unpredictable and heterogeneous nature of the disease. However, the individuality of this case lies within the elaborate series of occasions following surgical intervention. The development of a small bowel leak at the staple line, intra-stomach contamination, and next complications adds a layer of complexity no longer significantly explored in existing literature. While previous research acknowledges the potential want for surgery in Crohn's disorder, this example underscores the difficult interaction among chronic infection, postoperative complications, and the demanding situations in reaching a favorable outcome [10-12]. The nuanced details of this situation offer treasured insights into probable complications and emphasize the need for vigilant postoperative monitoring and timely intervention to optimize affected person consequences within the context of this difficult inflammatory disorder. Understanding such nuances is pivotal for refining healing strategies and improving standard patient care in the challenging landscape of Crohn's sickness [8,9].

Incorporating recent advancements into the management of CD is crucial for improving patient outcomes. A key aspect of preventing postoperative complications, such as leaks, is the effective coordination between emergency and scheduled surgeries. This approach minimizes the risk of adverse events by allowing for better preparation and planning. Additionally, recent advancements in endoscopic techniques, such as the placement of tubes for drainage through strictures, offer a promising alternative to emergency surgeries. This method can significantly reduce the risk of complications and improve overall patient management by providing a less invasive solution to intestinal obstructions. Emphasizing these advancements can enhance our understanding and approach to managing CD, ultimately leading to better patient care and outcomes [13].

In analyzing this case, it becomes evident that the development of tailored treatment plans, incorporating both established and novel therapeutic approaches, could greatly benefit future management of CD. Specifically, integrating advanced monitoring techniques and personalized care protocols could help anticipate and mitigate complications, ultimately improving patient outcomes. Emphasizing the role of timely interventions and careful postoperative management can enhance the efficacy of treatments and contribute to more successful long-term disease control. Future endeavors in CD management should focus on refining these strategies by leveraging emerging research on personalized medicine and the microbiome. Improved understanding and application of these approaches could potentially lead to better disease control and quality of life for patients, highlighting the importance of continued innovation and research in this challenging field.

## Conclusions

CD presents a multifaceted challenge due to its complex etiology involving genetic, environmental, and immune factors, affecting diverse demographics. This case study highlights the disease's heterogeneous nature and reveals critical gaps in understanding postoperative complications, such as small bowel leaks. These findings emphasize the need for vigilant postoperative monitoring and timely interventions to address and mitigate complications. Effective management of CD requires nuanced strategies to enhance patient care and therapeutic approaches, underscoring the importance of refining our understanding and treatment methods to improve outcomes and quality of life for patients.
